# Neurological impairment and disability in children in rural Kenya

**DOI:** 10.1111/dmcn.15059

**Published:** 2021-09-18

**Authors:** Jonathan A Abuga, Symon M Kariuki, Amina Abubakar, Christopher Nyundo, Samson M Kinyanjui, Michael Boele Van Hensbroek, Charles RJC Newton

**Affiliations:** ^1^ Department of Clinical Research (Neurosciences) Kenya Medical Research Institute‐Wellcome Trust Research Programme Kilifi Kenya; ^2^ Global Child Health Group Academic Medical Centre Emma Children’s Hospital University of Amsterdam Amsterdam the Netherlands; ^3^ Department of Psychiatry University of Oxford Oxford UK; ^4^ Institute for Human Development The Agha Khan University Nairobi Kenya; ^5^ Nuffield Department of Medicine University of Oxford Oxford UK

## Abstract

**Aim:**

To investigate geographical change over time in the burden of neurological impairments in school‐aged children in a demographic surveillance area.

**Method:**

We investigated changes in neurological impairment prevalence in five domains (epilepsy and cognitive, hearing, vision, and motor impairments) using similar two‐phase surveys conducted in 2001 (*n*=10 218) and 2015 (*n*=11 223) and determined changes in location‐level prevalence, geographical clustering, and significant risk factors for children aged 6 to 9 years (mean 7y 6mo, SD 1y) of whom 50.4% were males. Admission trends for preterm birth, low birthweight (LBW), and encephalopathy were determined using admission data to a local hospital.

**Results:**

Overall prevalence for any neurological impairment decreased from 61 per 1000 (95% confidence interval [CI] 48.0–74.0) in 2001 to 44.7 per 1000 (95% CI 40.9–48.6) in 2015 (*p*<0.001). There was little evidence of geographical variation in the prevalence of neurological impairments in either survey. The association between neurological impairments and some risk factors changed significantly with year of survey; for example, the increased association of adverse perinatal events with hearing impairments (exponentiated coefficient for the interaction=5.94, *p*=0.03). Annual admission rates with preterm birth (rate ratio 1.08, range 1.07–1.09), LBW (rate ratio 1.08, range 1.06–1.10), and encephalopathy (rate ratio 1.08, range 1.06–1.09) significantly increased between 2005 and 2016 (*p*<0.001).

**Interpretation:**

There was a significant decline in the prevalence of neurological impairments and differential changes in the associations of some risk factors with neurological impairments over the study period. Limited geographical variation suggests that similar interventions are appropriate across the defined area.

## Abstract 2

This original article is commented by Olusanya on pages 280–281 of this issue.

**Abbreviations**
KHDSS: Kilifi Health and Demographic Surveillance System
LBW: Low birthweight
LMIC: Low‐ and middle‐income countries
TQQ: Ten Question Questionnaire

**What this paper adds**

- Prevalence was reduced over time for childhood epilepsy and hearing and motor impairments.
- The association between adverse perinatal events and poor nutritional status and neurological impairments changed the most over the survey period.
- There was an increase over time in hospital admission rates with adverse pregnancy/birth outcomes.

Neurological impairments are an important cause of disability and premature mortality.^1^, ^2^, ^3^ At least 291 million (95% uncertainty interval 250–335) or 11.2% of all children and adolescents globally have epilepsy, intellectual disability, or sensory impairments, with the burden being higher in older children living in low‐ and middle‐income countries (LMIC).^4^ Sub‐Saharan Africa bears the highest prevalence of neurological impairments both in children under^5^ and above 5 years of age.^4^ Improved child survival^6^, ^7^ and increased incidence of known risk factors, such as adverse prenatal, perinatal, neonatal, and postnatal events, may have added to the prevalence of neurological impairments in older children in this region.^8^, ^9^ Africa is undergoing an epidemiological transition from infectious to non‐communicable diseases;^10^, ^11^ however, no current studies have investigated changes in the prevalence and risk factors of neurological impairments in older children over the period when control measures for infectious diseases were introduced.

There is a paucity of data on the prevalence of neurological impairments in children surviving the first 5 years of life in LMIC.^10^ A community survey conducted in rural Kenya in children aged 6 to 9 years in 2001 reported a prevalence of neurological impairments of 61 per 1000.^12^ This survey was done when malaria transmission was high,^13^, ^14^ before the introduction of vaccines against organisms causing meningitis, and when the health system was not as well developed as a decade later. Malaria control since 2001 has reduced the prevalence of malaria‐related seizure disorders including epilepsy;^15^, ^16^ however, the rate of admissions with neonatal risk factors, which may influence the prevalence of neurological impairments in the long term, increased during this period.^17^ Changes in the rate of hospital admissions with adverse neonatal events may reflect a general improvement in the utilization of maternal and childcare services in the community. However, the relative changes in prevalence due to the differential effects of these changing risk factors are yet to be examined in LMIC.

We hypothesized that the prevalence of neurological impairments in children aged 6 to 9 years would increase over a period of improved child survival characterized by a decline in mortality rates in children aged under 5 years, while recognizing that prevention of meningitis and malaria may decrease the neurological disability attributable to these infections. We also hypothesized that changes over time in the prevalence of neurological impairments from surveys conducted in a defined rural area might be associated with (directly or indirectly) or be consistent with secular trends of adverse neonatal events admitted to a hospital linked to the demographic surveillance system that produced the survey samples from 2005 to 2016. Therefore, we used the same methodology to conduct two successive epidemiological surveys in a defined demographic surveillance area, one in 2001 and another in 2015, to investigate geographical changes over time in the prevalence of and risk factors for neurological impairments and disability. We estimated the rates of neonatal admissions with preterm birth, neonatal encephalopathy, and low birthweight (LBW) using paediatric surveillance data from a rural referral hospital, which draws most of its admissions from the same defined area. Results from this study will provide evidence for setting research and policy priorities and planning public health interventions.

## METHOD

### Study setting

**Figure 1:**
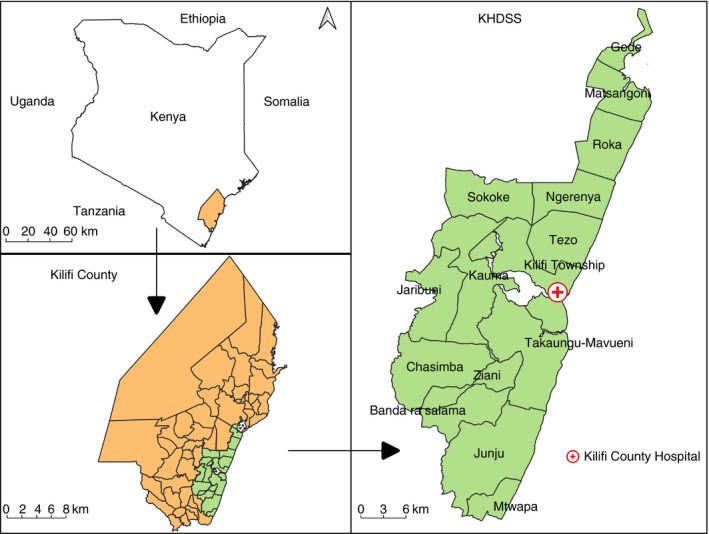
Map of Kenya showing the location of the Kilifi Health and Demographic Surveillance System (KHDSS) along the Kenyan coast.

We conducted epidemiological surveys and hospital surveillance of paediatric admissions in the Kilifi Health and Demographic Surveillance System (KHDSS), a defined geographical area covering approximately 900km^2^ in Kenya (Fig. 1). The KHDSS is re‐enumerated once every 4 to 6 months by a household survey to update vital statistics and indicators of socioeconomic status since 2000. The total resident population increased from 198 063 in 2001 to 279 158 in 2012, an increase mainly driven by high birth rates.^18^ Neonatal and under‐5 years mortality per 1000 live births are 17 and 41 respectively.^18^ Over 80% of inpatient admissions to Kilifi County Hospital originate from the KHDSS. This closely linked community, clinical, and hospital surveillance has been used to define the incidence and prevalence of various infectious and non‐infectious diseases.^13^, ^19^

We obtained approval for the epidemiological studies from the National Ethical Review Committee of the Kenya Medical Research Institute; permission to use data from the KHDSS was granted by the Data Governance Committee at the Kenya Medical Research Institute‐Wellcome Trust Research Programme in Kilifi. Written informed consent was sought from the parents, guardians, and caregivers of the participants in all phases before data collection.

### Epidemiological surveys

#### Study design

We used the same methodology to conduct two separate epidemiological surveys in the KHDSS to determine changes in the prevalence of and risk factors for neurological impairments in children aged 6 to 9 years. The justification for including children aged 6 to 9 years in these surveys is explained elsewhere.^1^ The first epidemiological survey was conducted between June 2001 and March 2002 and the second survey between March 2015 and January 2016. In these two‐phase surveys, a randomly selected sample of children aged 6 to 9 years (each with an equal probability of participating in the study) was screened for neurological impairment symptomatology in their households using the Ten Question Questionnaire (TQQ) (see Appendix S1, online supporting information),^12^, ^20^, ^21^ followed by a detailed clinical and neurological assessment to confirm the neurological impairment diagnosis. It was not feasible to conduct comprehensive clinical and neuropsychological assessments in the households; this necessitated a second phase, when selected participants from phase 1 were invited to the Neuroscience Unit of the Kenya Medical Research Institute‐Wellcome Trust Research Programme for detailed assessments.

#### Phase 1 (screening)

In phase 1, trained interviewers fluent in the local dialects administered the TQQ to the parents of the sampled children in their households. The selection of more than one child from the same household was rarely witnessed, despite each eligible child having an equal probability of selection. We trained the interviewers and pretested the TQQ until interobserver variability was less than 5% in both surveys before data collection. Data collected at this phase had household‐level geographical coordinates and each participant was assigned a unique identification number. All children with a positive response in at least one of the TQQ items and every fifth child screening negative in all the TQQ items were invited to the Neuro‐Assessment Unit at the Kenya Medical Research Institute‐Wellcome Trust Research Programme in Kilifi County Hospital for comprehensive assessment.

#### Phase 2 (detailed assessments)

Comprehensive clinical and neuropsychological assessments were conducted by a team of experts blinded to screening status in the second phase of each survey. A parental questionnaire was administered to the guardians/parents to obtain sociodemographic and medical history information, such as adverse pregnancy, birth and neonatal events, immunization, history of seizures, and previous hospitalization (nested case–control studies) after signing the consent form. Adverse perinatal events were defined as difficulties in breathing, feeding, or crying after delivery in both surveys. Children with a history of two unprovoked seizures had an electroencephalogram to classify the type of seizures and epilepsy. The Sonksen–Silver acuity system and calibrated audiometry were used to assess visual and hearing impairments respectively. Motor impairments were assessed by clinical examination using a standardized examination form.^12^ A seven‐item battery was used to test verbal and non‐verbal skills in the 2001 survey.^12^ In 2015, the Raven’s Coloured Progressive Matrices^22^ and Kilifi Naming Test^23^ were administered to evaluate non‐verbal skills and language/communication deficits respectively. We defined cognitive impairments using z‐scores from these neuropsychological scores standardized to the general population. We defined neurological impairments as the presence of at least one impairment in the five domains assessed (Table S1, online supporting information). We also measured the height and weight of every participant; nutritional status was based on weight‐for‐age and height‐for‐age z‐scores using the World Health Organization’s child growth charts as reference.

### Hospital surveillance

Prospective surveillance of paediatric admissions has been in place at Kilifi County Hospital since 1989. We reviewed all admissions occurring 28 days or less after delivery between January 2005 and December 2015. Clinical diagnoses at Kilifi County Hospital are determined after reviewing admission history, inpatient management notes, and laboratory investigations at the point of discharge.^17^ Preterm birth was defined as any neonate born before completing 37 weeks of gestation if the date of the mother’s last monthly period was known. If the date was unknown, the gestation week at delivery was estimated by the admitting clinician using a simplified criterion based on head circumference, mid‐upper arm circumference, breast size, ear form, genitalia, and skin texture.^24^ Neonatal encephalopathy was defined as any newborn infant having at least three of the following criteria: difficult delivery; signs of poor crying; convulsions; coma; irritability; or abnormal muscle tone.^8^ LBW was defined as newborn infants weighing 2500g or less according to the World Health Organization guidelines.

### Statistical analyses

Descriptive and inferential statistical analyses were done in R (R Foundation for Statistical Computing, Vienna, Austria). Missingness was small (<5%); thus, we analysed the available data, which were well powered to detect differences owing to the large sample size. We used a standardized method^25^ to calculate adjusted prevalence rates for the 2001 and 2015 surveys for direct comparisons. Crude prevalence was calculated by dividing the number of confirmed cases in phase 2 by the total number of participants screened in phase 1. Adjusted prevalence estimates were calculated to account for TQQ sensitivity and specificity and attrition proportions between the phases in the surveys. Since the 2001 and 2015 samples of children aged 6 to 9 years were large and independent, we used a two‐sample *z*‐test to test the null hypothesis that there was no difference in the proportion of children with neurological impairments between the 2001 and 2015 surveys.

The list of biologically plausible risk factors included in this analysis was based on expert opinion and published evidence from the literature.^26^ We fitted separate logistic regression models to assess the association between each selected modifiable risk factor and each neurological impairment domain as the outcome separately for each survey (i.e. 2001 and 2015). We then combined data from the two surveys and assessed two‐way interactions by year of survey for each risk factor separately. We checked all assumptions for binary logistic regression before reporting odds ratios (ORs) and 95% confidence intervals (CIs) for the main effects and two‐way interaction coefficients by year of survey.

In the spatial analysis, we used community survey data for all cases and controls whose geographical coordinates at the household level were verifiable. We applied the Bernoulli probability model^27^ in the SatScan software Version 9.6 (Department of Medicine, Harvard Medical School, Boston, MA, USA) to scan for areas with high and low rates of neurological impairments under purely spatial analyses. We used the default (<50%) maximum spatial cluster size for the population at risk and a circular window shape in the parameter settings. A high cluster was defined as a geographical area with a higher number of cases than would be expected by chance. We used QGIS Version 3.8 ‘Zanzibar’ (Open Source Geospatial Foundation, Chicago, IL, USA), an open‐source geographical information software program, to map the identified clusters and the estimated location‐level prevalence of neurological impairments.

Finally, we fitted Poisson regression models to evaluate trends over time in the annual neonatal admission rates for preterm birth, LBW, and encephalopathy between 2005 and 2015, using time as the predictor and the number of live births in the KHDSS as the denominator (offset). Estimates of the average annual percentage changes were determined from the fitted regression models.

## RESULTS

A total of 10 218 and 11 223 children aged 6 to 9 years were screened using the TQQ in 2001 and 2015 respectively; 810 (84.8%) out of 955 (9.3%) children with a positive TQQ result and 766 (89.0%) of 861 (9.3%) randomly sampled controls were assessed in phase 2 in 2001. In 2015, 70% of all children screening positive on the TQQ and the randomly selected controls were assessed in phase 2. A summary of survey characteristics comparing the 2001 and 2015 surveys is shown in Table 1. Similarly, adverse neonatal events between 2005 and 2016 in Kilifi County Hospital are shown in Table S2 (online supporting information).

**Table 1:** Survey characteristics and changes over time in the prevalence of neurological impairments in children aged 6 to 9 years in the KHDSS, Kenya, comparing the 2001 and 2015 epidemiological surveys

Study characteristic	2001 Neurological Impairment Survey	2015 Neurological Impairment Survey	*p*
Total population of children aged 6–9y in the KHDSS	25 250	37 294	–
Number of children screened using the TQQ tool in each household	10 218	11 223	–
TQQ sensitivity for moderate‐to‐severe impairments, % (95% CI)			
Epilepsy	100 (73.2–100)	86.7 (86.1–87.3)	–
Cognition	70.0 (63.2–76.0)	84.3 (83.6–84.9)	–
Hearing	87.4 (78.1–93.2)	85.7 (85.0–86.3)	–
Vision	77.8 (40.2–96.1)	100 (100–100)	–
Motor	71.4 (47.7–87.8)	93.7 (93.3–94.2)	–
Overall	81.5 (72.3–89.1)	86.5 (85.7–87.0)	–
TQQ specificity for moderate/severe impairments, % (95% CI)			
Epilepsy	92.9 (91.5–94.1)	84.7 (79.3–85.3)	–
Cognition	71.4 (69.1–73.6)	84.4 (83.7–85.0)	–
Hearing	85.5 (83.6–87.1)	84.1 (83.4–84.8)	–
Vision	98.0 (97.4–98.7)	84.1 (83.4–84.8)	–
Motor	98.3 (97.5–98.8)	84.2 (83.5–84.8)	–
Overall	84.2 (80.2–91.3)	84.5 (83.1–85.1)	–
Overall attrition between the two phases, %	15.2	30.0	–
Number of screen negatives who turned positive in phase 2, *n*	64	50	–
Prevalence per 1000 (95% CI), adjusted for TQQ sensitivity and attrition between phases			
Any impairment	61.0 (48.0–74.0)	44.7 (40.9–48.6)	<0.001
Epilepsy	41.0 (31.0–51.0)	17.2 (15.0–19.9)	<0.001
Cognition	31.0 (22.0–41.0)	27.1 (24.2–30.3)	0.089
Hearing	14.0 (9.0–18.0)	3.0 (2.1–4.2)	<0.001
Motor	5.0 (2.0–18.0)	2.7 (1.8–3.8)	0.006
Vision	2.0 (0–18.0)	1.8 (1.2–2.9)	0.737

KHDSS, Kilifi Health and Demographic Surveillance System; TQQ, Ten Question Questionnaire; CI, confidence interval.

Three hundred and six (3.0%) children out of 10 218 assessed in 2001 had neurological impairments; in 2015, 251 (2.2%) out of 11 223 children aged 6 to 9 years who were assessed had neurological impairments. The overall adjusted prevalence for any neurological impairment, adjusted for TQQ sensitivity and specificity and attrition between phases, decreased from 61.0 per 1000 (95% CI 48.0–74.0) in 2001 to 44.7 (95% CI 40.9–48.6) in 2015 (26.7% reduction; *p*<0.001). The prevalence of lifetime epilepsy was reduced from 41.0 per 1000 (95% CI 31.0–51.0) in 2001 to 17.2 (95% CI 15.0–19.9) in 2015 (*p*<0.001). Similarly, the prevalence of hearing impairments was reduced from 14.0 per 1000 (95% CI 9.0–18.0) in 2001 to 3.0 (95% CI 2.1–4.2) in 2015 (*p*<0.001). However, the prevalence of cognitive and visual impairments did not significantly change over the 14 years (Table 1).

**Figure 2:**
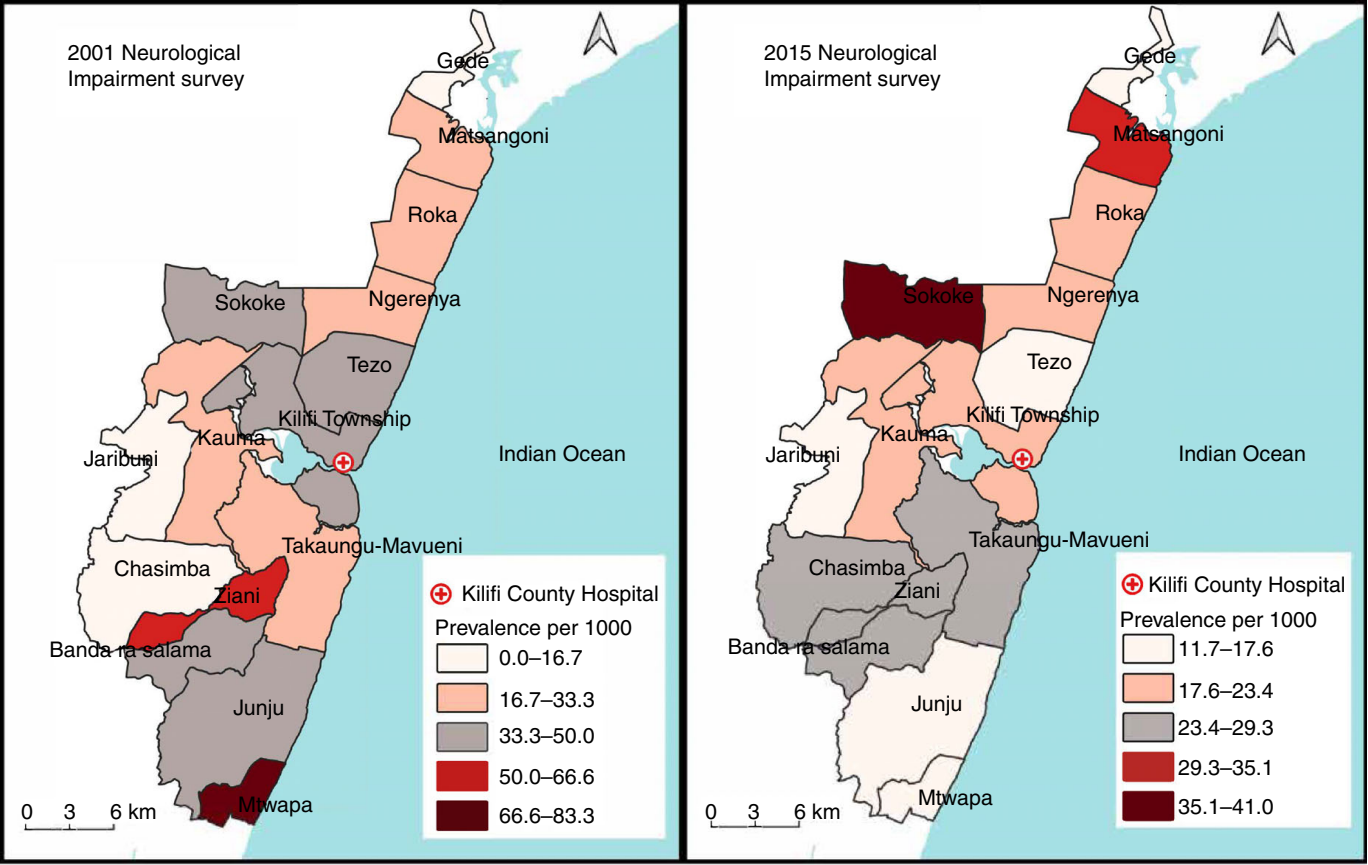
Map showing the changes in the prevalence of neurological impairments in children aged 6 to 9 years in the locations of the Kilifi Health and Demographic Surveillance System, Kenya, using data from the 2001 and 2015 surveys respectively. An equal interval method was used to define the ranges in QGIS symbology.

There was a significant decline in the prevalence of neurological impairments in children aged 6 to 9 years at four locations in the KHDSS, namely Kilifi township, Mtwapa, Tezo, and Ziani (Fig. 2). Actual data for changes in location‐level prevalence are provided in Table S3 (online supporting information). Generally, there was little evidence of geographical clustering at both survey points. In summary, four high and three low clusters and 10 high and four low clusters were identified in 2001 and 2015 respectively (Fig. S1, online supporting information). In 2001, only one low cluster was statistically significant (*p*=0.010) while one high cluster was statistically significant in 2015 (*p*=0.033).

Adverse perinatal events were significant predictors of epilepsy and cognitive and motor impairments in both surveys and for hearing impairment in 2015 (Tables 2 and 3); however, a significant increase over time in the strength of the association was observed only for hearing impairment by 2015 (exponentiated coefficient for the interaction=5.94, *p*=0.03) (Table 4). Higher weight‐for‐age and height‐for‐age z‐scores were inversely associated with motor and hearing impairments in 2001 and with cognitive impairments in both surveys (Tables 2 and 3). Notably, there was a significant decrease over time in the association of weight for age with cognitive and motor impairments (exponentiated coefficient for the interaction=0.49 and 0.95 respectively) and height for age with cognitive and motor impairments (exponentiated coefficient for the interaction=0.44 and 0.89 respectively; Table 4). Home delivery, atypical delivery, neonatal jaundice, incomplete immunization, previous hospitalization, and a family history of seizures were positively associated with one or more impairments in either survey (Tables 2 and 3). However, the odds of previous hospitalization were significantly higher only in children with epilepsy in 2001 (exponentiated coefficient for the interaction=2.69, *p*=0.04). Similarly, the odds of home delivery in children with cognitive impairment was higher in 2001 (exponentiated coefficient for the interaction=1.33, *p*=0.02).

**Table 2:** Comparison of the odds ratios (ORs) and 95% confidence intervals (CIs) by year of survey for the selected risk factors of epilepsy and cognitive and motor impairments in a defined rural setting, Kilifi, Kenya

Risk factor	Epilepsy		Cognitive impairment		Motor impairment	
	2001 (*n*=110)	2015 (*n*=98)	2001 (*n*=159)	2015 (*n*=148)	2001 (*n*=55)	2015 (*n*=18)
Atypical pregnancy	0.68 (0.44–1.09)	1.13 (0.65–1.87)	0.94 (0.63–1.45)	1.13 (0.72–1.72)	0.56 (0.31–1.05)	1.41 (0.40–3.97)
Home vs hospital delivery	0.65(0.41–1.09)	0.82 (0.53–1.31)	1.67 (1.01–2.98)	0.80 (0.56–1.17)	0.89 (0.45–1.98)	0.67 (0.26–1.93)
Birthweight	1.07 (0.65–1.66)	0.98 (0.72–1.05)	0.97 (0.53–1.65)	0.85 (0.59–1.04)	0.94 (0.42–1.85)	0.77 (0.33–1.09)
Atypical delivery	1.32 (0.65–2.43)	2.15 (1.28–3.46)	1.45 (0.82–2.45)	1.61 (1.01–2.46)	3.14 (1.50–6.05)	2.73 (0.87–7.29)
Adverse perinatal events	1.84 (1.14–2.87)	2.18 (1.16–3.81)	1.81 (1.19–2.68)	2.23 (1.33–3.59)	2.35 (1.25–4.21)	4.79 (1.52–12.89)
Neonatal jaundice	1.88 (1.01–3.33)	2.72 (1.24–5.36)	2.01 (1.17–3.30)	1.10 (0.42–2.36)	2.56 (1.15–5.15)	3.18 (0.50–11.45)
Incomplete immunization of the child	0.36 (0.09–1.05)	1.44 (0.43–3.61)	0.96 (0.46–1.82)	0.71 (0.17–1.96)	0.53 (0.08–1.81)	2.05 (0.11–10.29)
Family history of seizures	1.38 (0.80–2.28)	2.07 (1.32–3.18)	1.01 (0.61–1.61)	1.04 (0.68–1.56)	0.50 (0.15–1.25)	2.04 (0.71–5.28)
Weight for age (underweight)	1.01 (0.84–1.22)	0.98 (0.83–1.29)	0.61 (0.52–0.71)	0.86 (0.69–0.98)	0.54 (0.43–0.68)	0.84 (0.70–1.06)
Height for age (stunting)	0.93 (0.79–1.09)	1.02 (0.87–1.30)	0.59 (0.51–0.68)	0.89 (0.79–0.99)	0.48 (0.39–0.60)	0.83 (0.70–1.13)
Previous hospitalization	4.55 (2.98–7.12)	2.46 (1.63–3.70)	2.11 (1.51–2.95)	2.10 (1.48–2.95)	3.18 (1.81–5.77)	7.32 (2.75–22.90)

Data are OR (95% CI).

**Table 3:** Comparison of the odds ratios (ORs) and 95% confidence intervals (CIs) by year of survey for the selected risk factors of hearing and visual impairments in a defined rural setting, Kilifi, Kenya

Risk factor	Hearing impairment		Visual impairment	
	2001 (*n*=70)	2015 (*n*=18)	2001 (*n*=15)	2015 (*n*=14)
Atypical pregnancy	0.87 (0.49–1.61)	0.61 (0.10–2.17)	3.42 (0.68–62.15)	1.98 (0.54–5.96)
Home vs hospital delivery	0.93 (0.50–1.90)	1.18 (0.42–4.17)	^a^	0.45 (0.15–1.36)
Birthweight	1.02 (0.46–1.94)	^a^	^a^	^a^
Atypical delivery	1.09 (0.41–2.38)	1.62 (0.37–5.07)	0.82 (0.04–4.11)	0.58 (0.03–2.97)
Adverse perinatal events	0.93 (0.44–1.74)	3.81 (1.06–10.91)	0.38 (0.02–1.92)	3.36 (0.75–10.89)
Neonatal jaundice	1.40 (0.57–2.94)	^a^	1.90 (0.30–7.01)	^a^
Incomplete immunization	0.40 (0.06–1.36)	^a^	4.63 (1.16–16.53)	^a^
Family history of seizures	0.82 (0.36–1.64)	1.56 (0.50–4.17)	0.46 (0.03–2.30)	0.67 (0.10–2.48)
Weight for age (underweight)	0.77 (0.62–0.99)	1.22 (0.76–2.11)	0.77 (0.51–1.23)	1.36 (0.82–2.10)
Height for age (stunting)	0.82 (0.67–0.99)	1.30 (0.77–2.13)	0.76 (0.51–1.14)	0.83 (0.71–1.07)
Previous hospitalization	1.34 (0.82–2.18)	2.79 (1.08–7.17)	2.31 (0.83–6.94)	3.72 (1.29–11.33)

Data are OR (95% CI).

^a^ Small sample sizes prevented fitting a logistic regression model.

**Table 4:** Exponentiated coefficients for two‐way interactions by year of survey (2001 vs 2015) and the selected risk factors for the five domains of neurological impairment in a defined rural setting, Kilifi, Kenya

Risk factor	Epilepsy (*n*=208)		Cognitive impairment (*n*=307)		Motor impairment (*n*=73)		Hearing impairment (*n*=88)		Visual impairment (*n*=29)	
	Exponentiated coefficient for the interaction	*p*	Exponentiated coefficient for the interaction	*p*	Exponentiated coefficient for the interaction	*p*	Exponentiated coefficient for the interaction	*p*	Exponentiated coefficient for the interaction	*p*
Atypical pregnancy	0.78	0.48	1.59	0.85	6.79	0.72	5.54	0.43	0.57	0.11
Home vs hospital delivery	0.44	0.55	1.33	0.02	3.04	0.62	5.85	0.74	0.99	0.98
Birthweight	0.94	0.79	0.46	0.67	1.77	0.76	3.42	0.97	<0.01	0.99
Atypical delivery	1.27	0.30	2.39	0.96	14.06	0.75	7.34	0.67	1.27	0.79
Adverse perinatal events	1.45	0.57	2.38	0.31	9.74	0.22	5.94	0.03	0.68	0.07
Neonatal jaundice	1.84	0.42	2.60	0.29	9.71	0.76	6.99	0.97	2.51	0.99
Incomplete immunization of the child	0.76	0.08	1.80	0.68	2.63	0.28	2.48	0.99	6.15	0.99
Family history of seizures	1.14	0.15	1.38	0.69	2.52	0.06	4.74	0.27	0.62	0.72
Weight for age (underweight)	0.77	0.75	0.49	0.01	0.95	<0.01	3.92	0.30	0.45	0.11
Height for age (stunting)	0.93	0.52	0.44	<0.01	0.89	<0.01	4.72	0.19	0.72	0.74
Previous hospitalization	2.69	0.04	2.84	0.96	19.00	0.17	9.30	0.17	3.78	0.54

An exponentiated coefficient for the interaction >1 signifies an increase in the strength/direction of the association, while an exponentiated coefficient for the interaction <1 signifies a decrease.

A separate analysis of admissions to hospital showed that there was a significant increase in the admission rate of preterm birth (rate ratio 1.08, 95% CI 1.07–1.09, *p*<0.001), LBW (rate ratio 1.08, 95% CI 1.06–1.10, *p*<0.001), and neonatal encephalopathy (rate ratio 1.08, 95% CI 1.06–1.09 *p*<0.001) between 2005 and 2016 as assessed from admissions to Kilifi County Hospital (Fig. S2, online supporting information). The annual average percentage change in admission rate with preterm birth was +8.36% (95% CI 7.07–9.67); it was +8.55% (95% CI 7.02–10.10) for neonatal encephalopathy and +6.49% (95% CI 4.40–8.62) for LBW. The cumulative risk of preterm birth among newborn infants comparing the 4‐year periods between 2005 and 2016 is provided in Table S4 (online supporting information).

## DISCUSSION

The overall adjusted prevalence of neurological impairments did not increase as hypothesized but rather decreased from 61 per 1000 in 2001 to 44.7 per 1000 in 2015. This reduction was mainly driven by a decline in the prevalence of epilepsy, hearing, and motor impairments; the prevalence of cognitive and visual impairments did not significantly change over the 14‐year period. Adverse perinatal events and undernutrition were significant risk factors for neurological impairment in both surveys; other risk factors, such as place of delivery (2001 survey), immunization status (2001 survey), and a family history of seizures (2015 survey) were significant only in one survey. There was a significant change in the association of adverse perinatal events with hearing impairments (an increase) and undernutrition with motor impairments (a decrease) when we compared the 2001 and 2015 surveys. Generally, there was limited evidence of geographical variation in the prevalence of neurological impairments in both surveys. The rate of hospital admissions with adverse neonatal events increased significantly between 2005 and 2016, which could be related to improved utilization of hospitals and increased births in health facilities over the years.

The overall prevalence of neurological impairments was significantly reduced from 61 per 1000 to 44.7 per 1000 in 2015. This decline was driven, in part, by a decline in the prevalence of epilepsy (from 41.0 per 1000 to 17.2 per 1000). The 2001 Neurological Impairment Survey was conducted when malaria transmission was high^13^, ^14^ and the reduced prevalence of epilepsy may be attributed to a decline in seizures attributable to malaria and meningitis, which subsequently increased the risk for epilepsy.^15^, ^16^ The decline in the prevalence of epilepsy may also be attributed to sensitizations and prompt management of acute seizures, which reduced the risk of subsequent unprovoked seizures.^28^

The introduction of Haemophilus and pneumococcal vaccines might have reduced the overall burden of neurological impairments, such as hearing and epilepsy, by reducing the incidence of childhood meningitis. Second, the prevalence of hearing impairments decreased between the two surveys possibly due to a shift from the use of gentamicin (ototoxic effects^29^) to the use of cephalosporins/cefotaxime instead of aminoglycosides to treat bacterial infections in children. However, empirical studies are required to determine if changes in antibiotic use and the introduction of these vaccines are associated with a reduction in admissions to hospitals for children with neurological impairments. The decline in the prevalence of motor impairments is likely explained by the health benefits of an increased number of hospital deliveries in the years preceding the latter survey,^30^ although some would also reduce due to the control measures for infectious diseases described earlier, particularly Plasmodium falciparum malaria.^31^

The prevalence of cognitive and vision impairments did not significantly change between the two surveys; this could mean that known (and unknown) risk factors for these impairments, such as adverse perinatal and neonatal events, are unaddressed. Admissions with neonatal illnesses from a separate data set increased over the years;^17^ some were associated with individual domains of neurological impairment in either surveys in this and other studies.^32^, ^33^ Notably, the increased rate of hospital admissions in this data set might reflect a general improvement in health utilization in the community that may: (1) reduce the long‐term risk for neurological impairments from gains made by hospital management of these risk factors; and (2) indirectly improve the prognosis of neurological impairment if older children continue using hospital services as is the case for those with adverse perinatal events. However, a direct inference might be impossible because we are comparing different cohorts of children from different studies conducted at different time points. Visual impairments were associated with incomplete immunization in 2001, possibly because children with incomplete vaccine schedules did not receive vitamin A supplements or had vaccine‐preventable infections like measles in childhood;^34^, ^35^ however, other risk factors for neurological impairment driving high prevalence may have emerged.

Some risk factors were significantly associated with neurological impairments in both surveys. For instance, adverse perinatal events were positively associated with epilepsy and cognitive, motor, and hearing impairments. This is not a surprising finding because adverse perinatal events predispose newborn infants to hypoxia, which is an established cause of encephalopathy and other neurological sequelae.^36^, ^37^, ^38^ Also, the association between malnutrition and neurological impairment and disability is well established;^39^ thus, it is not surprising that higher weight‐for‐age and height‐for‐age z‐scores were protective against cognitive, motor, and hearing impairments. However, the effect sizes for the association between nutritional status and neurological impairment were reduced by 2015, which might reflect changes in the nutritional status of these children in the community. The other observed interactions by year of survey; for example, adverse perinatal events might be explained by changes in the utilization patterns of maternal and child health care services in Kilifi.

Some risk factors were significant in either of the two surveys, with others such as previous hospitalization being associated with most domains of neurological impairment in both surveys. Neonatal jaundice and incomplete vaccination were significantly associated with neurological impairment more in 2001 than in 2015, which may suggest that there was improved management of these conditions in subsequent years, including the 2015 survey period. Increased access and utilization of health care services might explain the importance of atypical deliveries in 2015. Family history of seizures was important in 2015, when there may have been better characterization and understanding of the phenotype, while lack of association in 2001 may underpin the increasing role of environmental–genetic interaction in later years. Some risk factors in both surveys could be inherently related to the nature of neurological impairments, for example, hospitalization where individuals with severe neurological impairment are expected to visit hospitals regardless of the survey period. Hospital admissions might be a consequence of neurological impairment since children with neurological impairments may seek out services, for example, patients with epilepsy seeking antiseizure medication, or related to neonatal conditions, such as jaundice and sepsis or encephalopathy.^40^

The rate of hospital admissions with preterm birth, LBW, and neonatal encephalopathy increased between 2005 and 2015. The observed increase may reflect improved utilization of health services or better reporting in the latter years and not necessarily an increase in the incidence of these conditions. While it is recognized that adverse perinatal events predispose to neurological impairment, we hypothesize that the increase seen in our hospital admissions does not directly affect the prevalence of reported neurological impairments because: (1) the hospital admission study did not start in 2001 when the baseline survey was conducted; and (2) participants in the hospital admission study are different from those participating in the surveys. It may require interventional and/or longitudinal studies to make a meaningful conclusion of the impact of such changes on the burden of neurological impairments. Besides, our hospital estimates may grossly underestimate the incidence of preterm birth and LBW, which are lower than elsewhere in sub‐Saharan Africa^41^ because a significant number of deliveries still occur at home, there is inadequate monitoring of deliveries occurring in hospitals in most LMIC,^8^ and more than half of the infants are not weighed at birth. However, our estimate for the annual admission with neonatal encephalopathy rate in 2010 (17.0 per 1000) is comparable with an estimated incidence rate of 14.9 per 1000 from studies conducted in similar settings.^8^

In the spatial analysis, the location‐level estimates of prevalence reflected the general decline observed in the larger KHDSS. However, the decline was significant in 4 out of 15 locations (Kilifi township, Mtwapa, Tezo, and Ziani). The observed change in Mtwapa might be a chance observation because there were very few participants from this location in 2001. The decline in the other three predominantly semi‐urban settings might represent locations that can easily access hospitals and are within reach of public health interventions. We also sought to identify areas with high or low clustering of neurological impairments in 2001 and 2015. Neurological impairments were generally homogeneously distributed in the KHDSS in both surveys except for one hotspot located towards the south of the KHDSS in 2015. Since the current study observed less geographical heterogeneity in the geographical distribution of neurological impairments, appropriate interventions on the significant risk factors may be applied evenly throughout the study area.

### Strengths and limitations of the study

We used the same methodology to conduct two large epidemiological surveys in a rural setting in the context of regular community and hospital surveillance, which is a rare resource in sub‐Saharan Africa. Intensive and comprehensive neuropsychological, clinical, and anthropological assessments and evaluations were carried out, which are usually unavailable in many LMIC. The analyses utilized data from an established KHDSS and linked hospital admissions data to estimate the trends in annual admission rates of adverse neonatal events. The interpretation of findings from all subanalyses in this study is contextually relevant since all participants were drawn from the same defined community. We used spatial analyses to identify high and low cluster areas for neurological disability, which may inform the choice of targeted or mass interventions.

Limitations in our study include: (1) a possibility for recall bias for medical history factors since we used a parental questionnaire in the surveys; (2) possible residual and unmeasured confounding that was beyond the scope of this study; (3) small sample sizes for some domains of neurological impairment, especially visual impairment, which reduced the power of the study to detect differences (possibility of type 2 errors); (4) the use of hospital data may have underestimated the rates of admissions with adverse pregnancy or birth outcomes because some neonates are not delivered in a hospital or die before they are admitted to the hospital (Neyman’s bias); (5) there were incomplete data to reliably examine adverse neonatal events before 2005; and (6) investigation of the factors explaining the clustering around the hotspot was beyond the scope of the current study. Further inquiry is required to identify specific factors explaining clustering around and within the single hotspot.

In summary, there was a significant decline in the prevalence of neurological impairments between 2001 and 2015 in Kilifi. A reduction in the prevalence of lifetime epilepsy and hearing impairments could be related to successes in the control of malaria‐attributable seizures, improved treatment of acute seizures, and other unevaluated interventions. The prevalence of cognitive and visual impairments did not change significantly. A decline in the prevalence of neurological impairments was significant in four locations; however, generally there was no geographical clustering. There was a significant change over time in the association of adverse perinatal events with hearing impairments and of undernutrition with cognitive/motor impairments. Analysis of hospital data showed that the rates of hospital admissions with adverse neonatal events significantly increased between 2005 and 2016.

## Supporting information


**Figure S1:** Clustering of neurological impairment in children aged 6 to 9 years in the KHDSS as identified by purely spatial scan analyses of data from the 2001 and 2015 surveys respectively.

**Figure S2:** Trends in annual admission rates with adverse perinatal and neonatal events based on the surveillance of paediatric admissions in Kilifi County Hospital between 2005 and 2016.

**Table S1:** Definitions of moderate and severe neurological impairments in the two surveys conducted in 2001 and 2015 respectively in Kilifi, Kenya

**Table S2:** Number of hospital admissions with adverse neonatal events between 2005 and 2016 in Kilifi County Hospital

**Table S3:** Comparison of the prevalence of neurological impairments in children aged 6 to 9 years in the locations of the KHDSS, Kenya, using data from the 2001 and 2015 neurological impairment surveys respectively

**Table S4:** Incidence rate ratio of preterm birth, low birthweight, and neonatal encephalopathy between 2005 and 2016 in the KHDSS

**Appendix S1:** The Ten Question Questionnaire.

## Data Availability

The data supporting our findings are available at the Havard Dataverse Repository (https://doi.org/10.7910/DVN/O2AX6C). Those interested in the data can apply through the data governance committee of the KEMRI‐Wellcome Trust Programme.
