# Supplementary figures and images for: Neurological impairment and disability in children in rural Kenya
Source: Dev Med Child Neurol. 2021 Sep 18;64(3):347–56. doi: 10.1111/dmcn.15059 (PMC9292953; doi:10.1111/dmcn.15059)

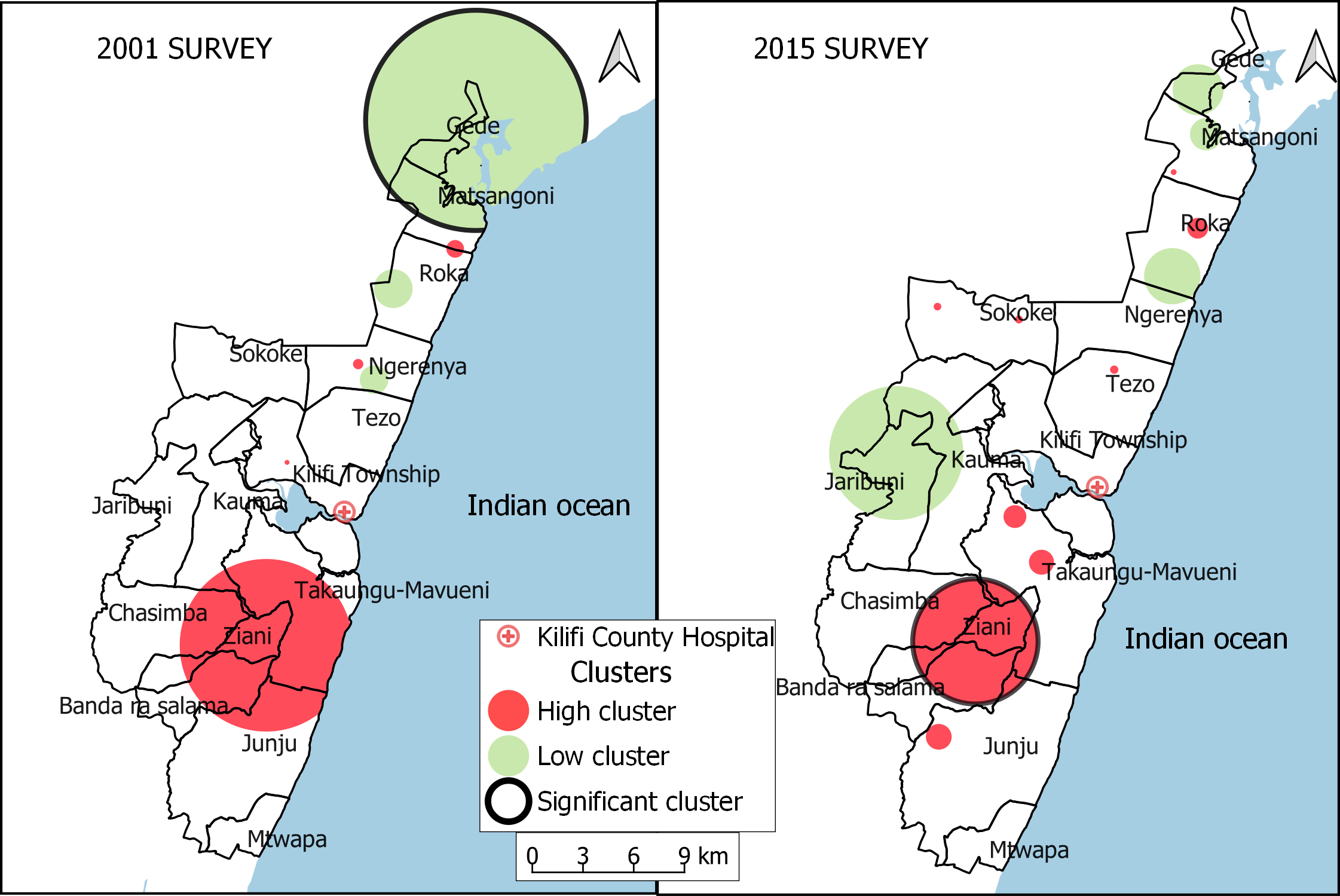

Supplement: Supplementary file 1 — Figure S1: Clustering of neurological impairment in children aged 6 to 9 years in the KHDSS as identified by purely spatial scan analyses of data from the 2001 and 2015 surveys respectively. [file DMCN-64-347-s002.tiff]

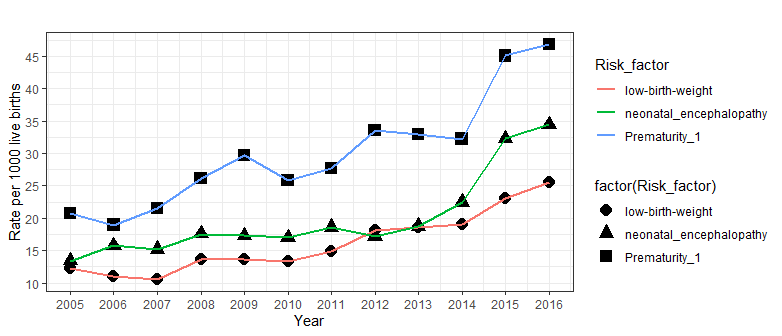

Supplement: Supplementary file 2 — Figure S2: Trends in annual admission rates with adverse perinatal and neonatal events based on the surveillance of paediatric admissions in Kilifi County Hospital between 2005 and 2016. [file DMCN-64-347-s005.tiff]
