# Supplementary material for: Neurological impairment and disability in children in rural Kenya
Source: Dev Med Child Neurol. 2021 Sep 18;64(3):347–56. doi: 10.1111/dmcn.15059 (PMC9292953; doi:10.1111/dmcn.15059)
Supplement: Supplementary file 3 — Table S1: Definitions of moderate and severe neurological impairments in the two surveys conducted in 2001 and 2015 respectively in Kilifi, Kenya [file DMCN-64-347-s004.docx]

**Supplementary Table 1: Definitions of moderate and severe neurological impairments in the two surveys conducted in 2001 and 2015, respectively, in Kilifi, Kenya**

| **Impairment** | **Moderate** | **Severe** |
| --- | --- | --- |
| Cognitive impairment | Z-score below -2 SD on the seven-item battery OR Raven’s Colored Progressive Matrices OR the Kilifi Naming Test. | Z-score below -3 on the seven-item battery OR Raven’s Colored Progressive Matrices OR the Kilifi Naming Test. |
| ^a^ Motor | Difficulty in holding items, dressing and sitting in an upright position; ambulant with help. | Immobile and no functional use of the hands. |
| ^a^ Hearing | A 41–70 dB loss in the best ear and difficulty in hearing with a hearing aid. | More than 70 dB loss in the best ear or complete deafness. |
| ^a^ Vision | Vision loss of 6/18 metres. | Visual acuity poorer than 6/60 metres and only light could be perceived. |
| ^b^ Epilepsy | More than one non-febrile seizure per month. | More than one non-febrile seizure per week. |

^a^ World Health Organization’s criteria used to define motor, hearing and vision impairments.

^b^ Epilepsy classified according to the International League Against Epilepsy’s (ILAE) criteria.
