# Supplementary material for: Neurological impairment and disability in children in rural Kenya
Source: Dev Med Child Neurol. 2021 Sep 18;64(3):347–56. doi: 10.1111/dmcn.15059 (PMC9292953; doi:10.1111/dmcn.15059)
Supplement: Supplementary file 4 — Table S2: Number of hospital admissions with adverse neonatal events between 2005 and 2016 in Kilifi County Hospital [file DMCN-64-347-s001.docx]

**Supplementary Table 2: Number of Hospital admissions with adverse neonatal events between 2005-2016 in Kilifi County Hospital**

| **Year** | **Neonatal admissions (percent)** | **Prematurity cases (percent)** | **Low-birth-weight (percent)** | **Neonatal encephalopathy**  **(percent)** | **Number of Live births in KHDSS** |
| --- | --- | --- | --- | --- | --- |
| 2005 | 717 (7.0) | 166 (5.8) | 98 (6.4) | 107 (5.6) | 7,992 |
| 2006 | 739 (7.2) | 154 (5.4) | 90 (5.9) | 128 (6.7) | 8,125 |
| 2007 | 688 (6.7) | 166 (5.8) | 81 (5.3) | 117 (6.2) | 7,701 |
| 2008 | 728 (7.1) | 204 (7.1) | 107 (7.0) | 137 (7.2) | 7,808 |
| 2009 | 778 (7.6) | 235 (8.2) | 108 (7.3) | 137 (7.2) | 7,919 |
| 2010 | 821 (8.0) | 219 (7.6) | 113 (7.3) | 144 (7.6) | 8,484 |
| 2011 | 873 (8.5) | 234 (8.2) | 125 (8.1) | 157 (8.3) | 8,444 |
| 2012 | 865 (84.) | 263 (9.2) | 142 (9.2) | 135 (7.1) | 7,845 |
| 2013 | 787 (7.7) | 267 (9.3) | 151 (9.8) | 152 (8.0) | 8,111 |
| 2014 | 953 (9.3) | 277 (9.7) | 163 (10.6) | 193 (10.1) | 8,594 |
| 2015 | 1,112 (10.8) | 342 (11.9) | 175 (11.4) | 245 (12.9) | 7,591 |
| 2016 | 1,200 (11.7) | 340 (11.9) | 185 (12.0) | 250 (13.1) | 7,258 |
| Total | 10,261 | 2,867 (100) | 1,538 (100) | 1,902 (100) | 95,872 |

KHDSS: The Kilifi Health and Demographic Surveillance System
