# Supplementary material for: Neurological impairment and disability in children in rural Kenya
Source: Dev Med Child Neurol. 2021 Sep 18;64(3):347–56. doi: 10.1111/dmcn.15059 (PMC9292953; doi:10.1111/dmcn.15059)
Supplement: Supplementary file 5 — Table S3: Comparison of the prevalence of neurological impairments in children aged 6 to 9 years in the locations of the KHDSS, Kenya, using data from the 2001 and 2015 neurological impairment surveys respectively [file DMCN-64-347-s007.docx]

**Supplementary Table 3: A comparison of the prevalence of neurological impairments in children aged 6-9 years in the locations of the Kilifi Health and Demographic Surveillance System, Kenya, using data from the 2001 and 2015 NI surveys, respectively.**

|  | **2001 NI survey** | | | **2015 NI survey** | | | **Difference in prevalence** | |
| --- | --- | --- | --- | --- | --- | --- | --- | --- |
| Location | Cases  N=306 | Survey  N=10,218 | Prevalence/1,000 (95%CI) | Cases  N=251 | Survey  N=11,223 | Prevalence/1,000  (95%CI) | % change | p-value |
| Banda ra salama | 6 | 128 | 46.9 (19.2-99.2) | 12 | 482 | 24.9 (13.5-44.3) | -46.89 | 0.192 |
| Chasimba | 0 | 61 | 0.0 (0-58.7) | 21 | 801 | 26.2 (16.7-40.0) | - | 0.200 |
| Gede | 4 | 519 | 7.7 (2.5-21.0) | 4 | 342 | 11.7 (3.75-31.7) | 51.75 | 0.551 |
| Jaribuni | 0 | 5 | 0.0 (0-521.8) | 4 | 263 | 15.2 (4.88-41.1) | - | 0.781 |
| Junju | 1 | 27 | 37.0 (1.9-209.0) | 20 | 1,383 | 14.5 (9.1-22.7) | -60.95 | 0.338 |
| Kauma | 5 | 276 | 18.1 (6.7-44.2) | 9 | 486 | 18.5 (9.1-36.1) | 2.22 | 0.968 |
| Kilifi township | 38 | 899 | 42.3 (30.5-58.1) | 14 | 682 | 20.5 (11.7-35.1) | -51.44 | 0.016 |
| Matsangoni | 29 | 1,250 | 23.2 (15.9-33.6) | 26 | 742 | 35.0 (23.5-51.6) | 51.04 | 0.119 |
| Mtwapa | 1 | 12 | 83.3 (4.4-402.0) | 6 | 507 | 11.8 (4.8-26.9) | -85.80 | 0.034 |
| Ngerenya | 38 | 1,342 | 28.3 (20.4-39.1) | 21 | 897 | 23.4 (14.9-36.2) | -17.32 | 0.478 |
| Roka | 35 | 1,309 | 26.7 (19.0-37.4) | 17 | 888 | 19.1 (11.6-31.1) | -28.40 | 0.251 |
| Sokoke | 19 | 541 | 35.1 (21.9-55.3) | 20 | 488 | 41.0 (25.9-63.7) | 16.70 | 0.623 |
| Takaungu/ Mavueni | 16 | 573 | 27.9 (16.6-45.9) | 33 | 1,158 | 28.5 (20.0-40.2) | 2.06 | 0.946 |
| Tezo | 55 | 1,608 | 34.2 (26.1-44.6) | 24 | 1,389 | 17.3 (11.3-26.0) | -49.48 | 0.004 |
| Ziani | 17 | 305 | 55.7 (33.8-89.4) | 20 | 712 | 28.1 (17.7-43.8) | -49.60 | 0.031 |
