# Supplementary material for: Neurological impairment and disability in children in rural Kenya
Source: Dev Med Child Neurol. 2021 Sep 18;64(3):347–56. doi: 10.1111/dmcn.15059 (PMC9292953; doi:10.1111/dmcn.15059)
Supplement: Supplementary file 6 — Table S4: Incidence rate ratio of preterm birth, low birthweight, and neonatal encephalopathy between 2005 and 2016 in the KHDSS [file DMCN-64-347-s003.docx]

**Supplementary Table 4: Incidence Rate Ratio (IRRs) of prematurity, low-birth-weight and neonatal encephalopathy between 2005-2016 in the Kilifi Health & Demographic surveillance system.**

|  | **Prematurity** | | **Low-birth-weight** | | **Neonatal encephalopathy** | |
| --- | --- | --- | --- | --- | --- | --- |
| **Period** | **IRR (95%CI)** | **p-value** | **IRR** | **p-value** | **IRR** | **p-value** |
| 2009-2012 Vs 2005-2008 | 1.40 (1.26-1.56) | <0.001 | 1.26 (1.10-1.44) | 0.001 | 1.13 (1.00-1.28) | 0.042 |
| 2013-2016 Vs 2005-2008 | 1.82 (1.65-2.02) | <0.001 | 1.80 (1.58-2.04) | <0.001 | 1.72 (1.54-1.92) | <0.001 |
| 2009-2016 Vs 2005-2008 | 1.61 (1.47-1.76) | <0.001 | 1.52 (1.35-1.71) | <0.001 | 1.42 (1.28-1.58) | <0.001 |
