# Supplementary material for: Neurological impairment and disability in children in rural Kenya
Source: Dev Med Child Neurol. 2021 Sep 18;64(3):347–56. doi: 10.1111/dmcn.15059 (PMC9292953; doi:10.1111/dmcn.15059)
Supplement: Supplementary file 7 — Appendix S1: The Ten Question Questionnaire. [file DMCN-64-347-s006.doc]

# **NEUROLOGICAL IMPAIRMENT SURVEY**

**THE TEN QUESTIONS (TQ)**

## Child’s Personal Details

# Today’s Date: …………………………………………………..… [__]__]/[__]__]/[__]__]__]__]

TQ Number: ………………………………………………………………….….. [__]__]__]__]__]

Child’s Name: ___________________________________________________________________

Mother’s Name: __________________________________________________________________

EZHHID: ……………………………………………………………… [__]__]__]__]__]__]__]__]

RESID: .…...…………………………………………………………………….… [__]__]__]__]__]

Child’s DOB: ……………………………………………………..... [__]__]/[__]__]/[__]__]__]__]

Child’s Age: ……………………………………………………………………………….. [__][__]

Child’s Sex: …………………………………………………………………………………….. [__]

Child’s place of Birth: ………………………………………………………………………….. [__]

Does the child attend school regularly? (Y/N) …………………………………………………. [__]

Who will answer questions about the child? ………………………………………………..….. [__]

1. The child’s mother. 2. The child’s father.

3. The child’s grandmother. 4. The child’s sibling.

5. Another relative. 6. Other.

Is the informant one who mainly takes care of the child?(Y/N) …………………………………[__]

Fieldworker Code…………………………………………………………………………………[__]

Child’s EZHHID: [__]__]__]__]__]__]__]

**Ten Questions**

Question 1. **Compared with other children, did the child**

**have any serious delay in sitting,**

**standing or walking?**  YES [__]* NO[__]

*Kuhalanisha na ahoho angine, yuno mwanao*

***Watoa sana*** *kukeresi, kuima koko hedu kunenda?*

______________________________________________________________________________

Question 2. **Compared with other children does the**

**child have difficulty seeing, either in**

**the daytime or at night?** YES [__]* NO [__]

*Kuhalanisha na ahoho angine, yuno muhoho*

*ana thabu ya kuona;*

*a] mtsana*

*b] hedu usiku?( kala kaona, mpaka agwirwe mkono?)*

_______________________________________________________________________________

Question 3. **Does the child appear to**

**have difficulty hearing?** YES [__]* NO [__]

*Yuno mwanao anathabu ya kusikira (hedu ana masikiro maziho)*

Question 4. **When you tell the child to do something,**

**does he/she seem to understand**

**what you are saying?**  YES [__] NO [__]*

*Ukimwambira utu, yuno mwanao nikuelewa*

*zho udzizho nena?*

_____________________________________________________________________________

Question 5. **Does the child have difficulty in walking or moving**

**his/her arms or does he/she have weakness and/ or**

**stiffness in the arms or legs?** YES [__]* NO [__]

Child’s EZHHID: [__]__]__]__]__]__]__]

*Yuno muhoho anathabu ya kunenda hedu kuusa*

*mikono hedu kukosa nguvu na/ hedu kumalala*

*mikono hedu magulu?*

_____________________________________________________________________________

Question 6. **Does the child sometimes have fits, become rigid,**

**Or lose consciousness?**  YES [__]* NO [__]

*Yuno mwanao nikufitika wakathi mungine akamalala*

*hedu kungamiza fahamu?*

________________________________________________________________________________

Question 7. **Does the child learn to do things**

**like other children his/her age?** YES [__] NO [__]*

*Yuno mwanao nikudzifundisha kuhenda*

*mautu here ahoho a marikage (here viryahu, kuheka madzi, kurisa hedu kushera)?*

________________________________________________________________________________

Question 8. **Does the child speak at all (can he/she make himself/**

**herself understood in words: can he/she say any**

**recognizable words)?**  YES [__] NO [__]*

*Yuno mwanao anadima kunena (anadima kunena*

*akaeleweka: anadima kunena maneno ga kumanyikana****)?***

________________________________________________________________________________

Question 9. **Is the child’s speech in any way different**

**from normal (not clear enough to be**

**understood by people they don’t talk to often)?** YES [__]* NO [__]

*Kuno kunenakwe yuno mwanao kunatofauti yoyosi (nikukala kaeleweka kwa atu ambao kamanena mara kwa mara) hedu ana kitsembe ama shida nyingine yoyosi ya kunena?*

________________________________________________________________________________

Child’s EZHHID: [__]__]__]__]__]__]__]

Question 10. **Compared with other children of his/her age**

**does the child appear in any way mentally**

**backward, dull or slow?** YES [__]* NO [__]

***Kuhalanisha na ahoho angine a rikare yuno mwanao***

***anathabu ya akili punguani, uzuzu hedu a goigoi?***

**Does the child have any serious health problem**

**not yet mentioned?** YES [__] NO [__]

*Yuno muhoho anathabu yoyosi ya kiafya iriyo kafwihadzire?*

If yes, write down what kind of problem it is:

________________________________________________________________________________

**Interviewer**: Answer the question below by circling of the three points. The questionnaire result

positive if the response to any one or more of the Ten Questions has an asterisk (*) next to it. If no

response has * next to it, then the result is negative.

**Should this child be referred for**

**professional evaluation**? YES [__] NO [__]

1. No, because the questionnaire result is negative

And there is no **x** in the box below.

1. Yes, because, although the questionnaire result is negative,

There is an **x** in the box below.

1. Yes, because the questionnaire result is positive.

For data entry only:

Does the box below contain an **x**? YES [__] NO [__]

[__]
